# Outcomes After Thrombectomy for Primary and Secondary Medium Vessel MCA Occlusions: a Nationwide Registry Study

**DOI:** 10.1007/s00062-025-01511-w

**Published:** 2025-03-31

**Authors:** Björn M. Hansen, Emma Hall, Birgitta Ramgren, Teresa Ullberg, Johan Wassélius

**Affiliations:** 1 Department of Medical Imaging and Physiology, Skåne University Hospital, Lund, Sweden; 2 Department of Clinical Sciences Lund, Radiology, Lund University, Lund, Sweden; 3 Department of Neurology, Skåne University Hospital, Malmö, Sweden; 4 Department of Clinical Sciences Lund, Neurology, Lund University, Lund, Sweden

**Keywords:** Stroke, Ischemic stroke, Endovascular thrombectomy, Medium vessel occlusion, Functional outcome, Secondary occlusion, Middle cerebral artery

## Abstract

**Background:**

Medium vessel occlusions (MeVO) can be either isolated events (primary), or secondary to thrombus migration from a large vessel occlusion to a medium-sized vessel. Outcomes following endovascular thrombectomy (EVT) in the middle cerebral artery (MCA) may differ between primary and secondary MeVOs. This study aimed to assess the association between primary/secondary MeVOs and clinical outcomes following EVT in a nationwide patient cohort.

**Method:**

Patients undergoing EVT were included in two Swedish quality registries. Secondary MeVO was defined as distal migration of a solitary thrombus between baseline CT-angiography and EVT, or basal ganglia infarction on postoperative CT in a patient that presented with a single MeVO on baseline CT-angiography. The primary outcome was good 90-day functional outcome (modified Rankin Scale 0–2). Postoperative change in the National Institutes of Health Stroke Scale-score (NIHSS), was a secondary outcome. Successfully revascularized patients (mTICI 2b–3) were compared with non-revascularized patients in exploratory analyzes.

**Results:**

Of the 5662 EVTs performed in Sweden (2018–2022), 1118 (20%) targeted solitary MCA territory MeVOs, with 819 (73%) being primary and 299 (27%) secondary. Functional outcomes did not differ between the primary and secondary MeVO groups (OR 0.86, CI 95% 0.65–1.14). Likewise, there was no significant difference in postoperative NIHSS scores (0.26, CI 95% −0.71 to 1.24), between groups (*p* = 0.597). Successful revascularization was associated with increased chance of good functional outcome for both primary (OR 3.77, CI95% 2.28–6.24, *p* < 0.001) and secondary MeVOs (OR 2.49, CI95% 1.21–5.14, *p* = 0.013).

**Conclusions:**

Patients with a single primary or secondary MCA MeVOs have similar EVT outcomes and both groups seem to benefit from recanalization in exploratory analyses. This indicates that that EVT should not be withheld based on primary/secondary MeVO status.

**Supplementary Information:**

The online version of this article (10.1007/s00062-025-01511-w) contains supplementary material, which is available to authorized users.

## Introduction

Endovascular thrombectomy (EVT) is now well-established as standard of care for large vessel occlusions (LVO) in the middle cerebral artery (MCA) territory [1, 2]. Indications for EVT in acute ischemic stroke have gradually widened. The next frontiers for EVT include patients with occlusion of a medium sized segment of the MCA, which accounts for one fourth of all ischemic stroke patients with visible arterial occlusion [3]. Several ongoing randomized controlled trials are currently evaluating the efficacy of EVT for medium vessel occlusions (MeVOs) compared to medical management [4]. It has been estimated that 20–40% of MeVOs result from an LVO that has been distally displaced, either spontaneously or as a result of treatments such as intravenous thrombolysis (IVT) [5]. Patients with secondary MeVOs may have more severe baseline stroke symptoms due to injuries from the initial LVO which are unlikely to be affected by revascularization of the MeVO [6]. Secondary MeVOs have been associated with worse outcomes after EVT compared to primary MeVOs in a recent observational study [7]. In contrast, a metanalysis of 14 studies showed similar outcomes for patients with primary and secondary MeVOs [8]. Hence, the effects of successful EVT in patients with primary and secondary MeVOs need to be studied further in broad patient samples representing real-world practice.

### AIMS

To analyze early neurological change and functional outcome at 3 months in patients with primary or secondary MCA territory MeVOs treated by EVT in a nationwide patient cohort.

## Methods

### Study Design

We conducted a nationwide register-based observational study based on data from the Swedish EndoVAscular treatment of Acute Stroke (EVAS) registry [9] and the Swedish national quality registry for stroke care, Riksstroke [10], collected between 2018 and 2022. The study was approved by the Swedish Ethical Review Authority (DNR 2019-00678 and 2022-00890-02).

### Patient Selection

Information on stroke characteristics such as occlusion location, procedural and treatment details, and postoperative complications, and pre- and post-treatment stroke severity were obtained from EVAS. EVAS continuously collect data from all of Sweden’s seven comprehensive stroke centers and has had a consistent national coverage above 98.5% of all EVT procedures performed during the study period when compared with the National Patient Register [9]. Healthcare professionals, including coordinators, neurointerventionalists, and diagnostic radiologists at each stroke center, report patient data to EVAS, each handling specific clinical and procedural information while blinded for the 90-day functional outcome assessment. Data on clinical baseline characteristics and 90-day functional outcome were obtained from Riksstroke which combine data from all 72 hospitals in Sweden managing acute stroke care (coverage of > 90%) [10]. Riksstroke’s standardized 90-day follow-up questionnaire for activities of daily life is self-reported by the patient or their next-of-kin or caregiver and was converted into the modified Rankin scale (mRS) using a validated algorithm [11]. Additionally, Riksstroke incorporates information on survival status from the National Cause of Death Register. The complementary information in the registers were linked using the official personal identity numbers that are assigned to all persons who are residing in Sweden and that are kept for life [12]. In patients with more than one EVT within 3 months only the first EVT was included in the study as the majority of re-treatments occurs within 7 days of the initial EVT [9] and might be regarded as a subacute complication of the primary treated stroke.

### Medium Vessel Occlusion (MeVO) Classification

The middle cerebral artery (MCA) segments were categorized as either M1, M2, or M3 and beyond, based on the reporting neurointerventionist’s description of the occlusion location in the MCA trajectory through the Sylvian triangle. The M2 segment is commonly defined by MCA turn in the insular region [13]. In this study occlusions in the MCA’s M2 or M3-segments were classified as MeVO, based on the proximal end of the thrombus on digital subtraction angiography (DSA) prior to EVT.

### Primary and Secondary MeVo Categorization

Patients with MCA territory MeVOs on both the initial computer tomography angiography (CTA) and the subsequent DSA were classified as *primary MeVOs*.

*Secondary MeVO* was defined as a single occlusion of the M2-segment or beyond on DSA, that originated as an occlusion in the internal carotid artery (ICA) or the MCA M1-segment on the initial CTA. Additionally, patients with new basal ganglia infarcts on postoperative computer tomography (CT) follow-up after EVT for MCA MeVO were also categorized as secondary MeVOs, since these infarcts are unlikely to have been caused by the treated M2/M3 segment occlusion indicating that a more proximal occlusion was dislodged prior to the baseline CTA. Patients with missing baseline CTA and no basal ganglia infarct on postoperative CT were excluded since primary/secondary status could not be assessed for them. Patients with multiple intracranial occlusions on DSA were excluded, as were patients with partial recanalization of the distal ICA or M1 segments and concomitant M2/M3 segment occlusions since we assumed that the partial large vessel occlusion would have been the primary target for treatment.

### Successful and Unsuccessful Revascularization

Successful revascularization was defined as a modified Thrombolysis in Cerebral Infarction (mTICI) score of 2b–3 on DSA after EVT [12]. EVT procedures with a mTICI score of 0–2a at the end of the procedure, or if EVT could not be performed due to inability to access the occlusion site, were defined as unsuccessful.

### Primary and Secondary Outcomes

The primary outcome measure was functional status at 90 days post-ictus using the modified Rankin Scale (mRS) with a good outcome defined as mRS 0–2. Patients with pre-stroke mRS 3–5 were excluded in this study. Early neurological improvement, measured as the absolute change in National Institutes of Health Stroke Scale (△NIHSS) scores before EVT and 24 h after, was a secondary outcome.

### Symptomatic Intracranial Hemorrhage

All intracranial hemorrhages on the 24-hour postoperative CT were classified as postoperative hemorrhagic complications. Symptomatic intracranial hemorrhage (sICH) was defined as any intracranial hemorrhage on the 24-hour postoperative CT accompanied by a severe neurological deterioration (≥ 4 NIHSS points) at 24-hours compared to the preoperative assessment, in accordance with the European Cooperative Acute Stroke Study (ECASS-III) criteria [13]. The ECASS-III sICH criteria also incorporate 7‑day case fatality, but these data were not available.

### Statistics

Descriptive analyses were done using Pearson’s χ2 test, Fisher’s exact test, and Mann-Whitney U test where appropriate. Two-sided *p*-values <0.05 were regarded as statistically significant. Logistic regression analyses were used for univariate and multivariate analyses, adjusting for age, sex, M3 segment occlusion (using M2 segment occlusions as reference), pre-stroke mRS, and baseline NIHSS, with 90-day good functional outcome (mRS 0–2) as dependent variable. T‑test and ANOVA (adjusting for age and sex) were used to assess change in stroke symptom severity before and after EVT (△NIHSS).

In exploratory outcome analyses patients were stratified by primary/secondary MeVO status and outcomes for recanalized patients were compared with non-recanalized patients. Patients with perioperative vessel perforation may have been overrepresented among non-revascularized patients due to the possibility of perforation leading to EVT termination. To address this, a sensitivity analysis excluding patients with sICH regardless of recanalization status was performed. In additional exploratory logistic regression analyses the baseline NIHSS was used as a surrogate for the anatomic location of the occlusion within the MCA vascular territory to investigate whether the treatment effect of EVT differs for patients with occlusions of the distal M2/M3 segments and proximal M2 segment occlusions. To do so groups were stratified based on preoperative stroke severity (preoperative NIHSS quartiles 1–2 and 3–4) and primary/secondary MeVO-status.

Multiple imputations by chained equations with age, sex, pre-stroke mRS and stroke severity before EVT as predictive values were used to account for missing data on functional outcome at 90 days for patients that were known to be alive at that time-point. Twenty complete datasets were constructed and the estimates from each imputation were combined using Rubin’s rule. The primary outcome analysis was also performed on the original dataset prior to multiple imputations.

## Results

**Fig. 1:**
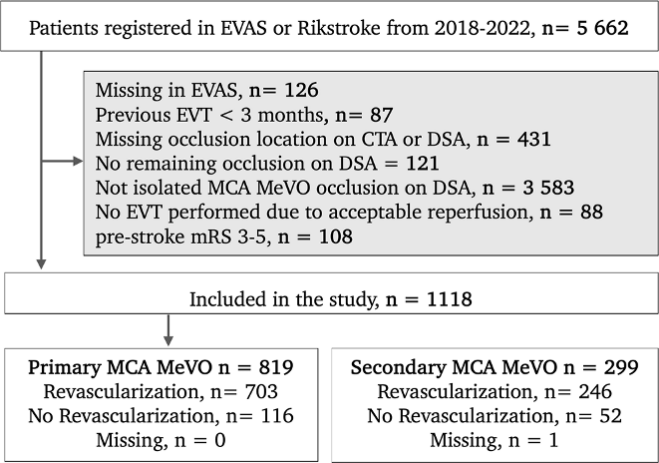
Flow-chart illustrating patient selection. Patients were included from the Swedish EndoVAscular treatment of Acute Stroke (EVAS) registry and the Swedish national quality registry for stroke care, Riksstroke. *EVT* Endovascular thrombectomy; *MeVO* Medium Vessel Occlusion; *MCA* Middle Cerebral Artery; *CTA* Computer Tomography Angiography; *DSA* digital subtraction angiography; *mRS* modified Rankin Scale

**Fig. 2:**
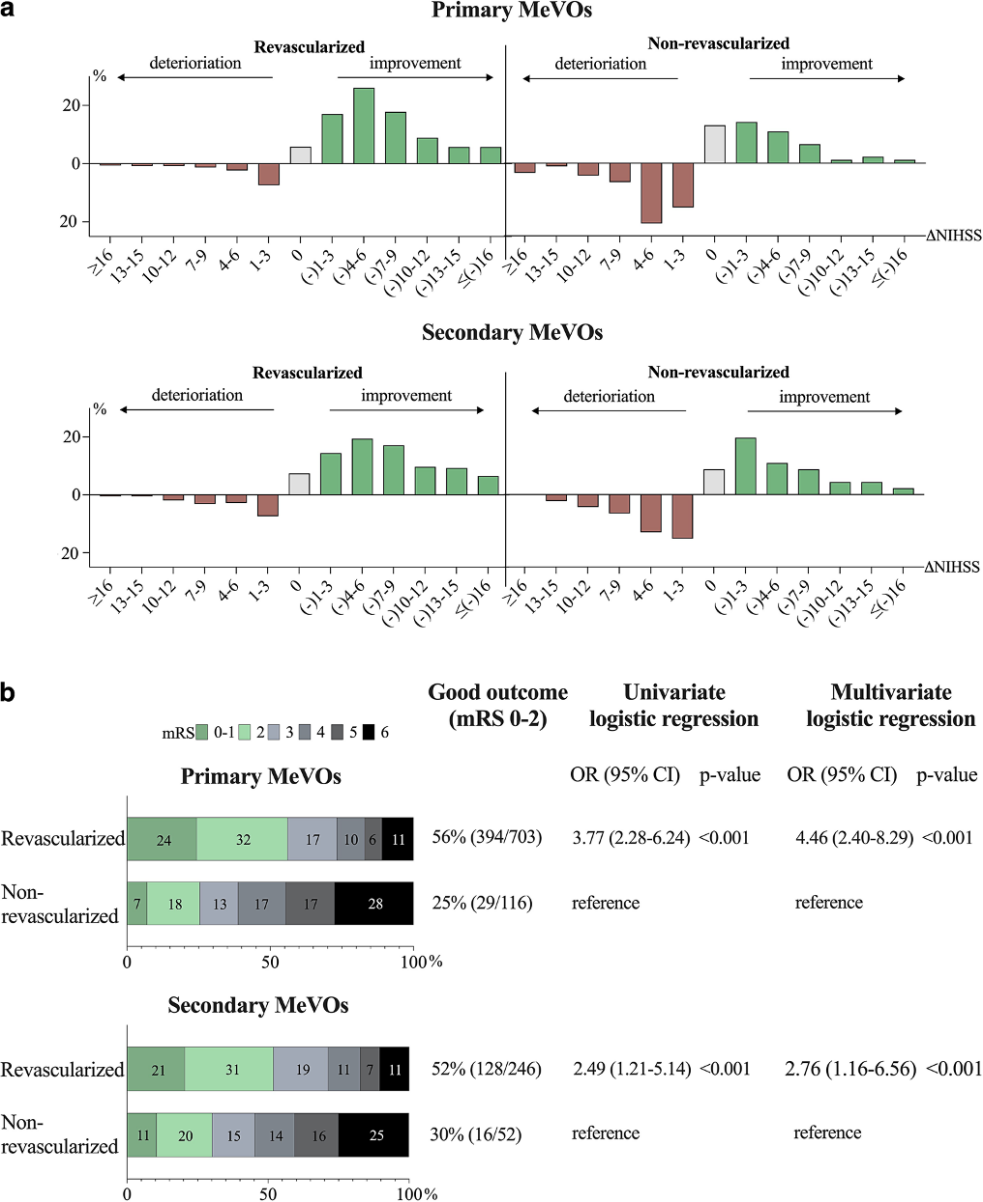
**a** Distribution of change in National Institutes of Health Stroke Scale before and 24 h after EVT (△NIHSS); **b** 90-day functional outcomes according to modified Rankin Scale (mRS); **c** results from logistical regression for good functional outcome (mRS 0–2), for patients with primary (*N* = 819) and secondary (*N* = 298) medium vessel occlusion (MeVO) based on revascularization status (mTICI 2b–3)

Of 5662 registered EVT procedures in EVAS or Riksstroke (2018–2022) 1118 patients (20%) had EVT for MCA territory MeVOs, of which 819 had primary (73%), and 299 had (27%) secondary MeVOs, respectively. A flow chart of the study population is shown in Fig. 1. Revascularization status was missing for one patient with a secondary MeVO. Data on functional outcome was missing for 285 (25%) patients that were known to be alive at 90-day follow-up, of which 214 (26%) had primary MeVOs and 71 (24%) secondary MeVOs. The distribution of 90-day functional outcomes mRS for patients with primary and secondary MeVOs stratified by revascularization status are shown in Fig. 2b.

### Primary and Secondary MeVo Groups

Among the 299 patients with secondary MeVOs, 190 had an M1 occlusion and 20 a terminal ICA occlusion on the initial CTA, the remaining 89 patients were characterized as secondary based on having new basal ganglia infarcts on the follow-up CT 24 h after EVT for an MCA MeVO. In both groups 12% of the patients had M3 segment occlusions (primary *N* = 36/299; and secondary *N* = 99/819) while the remaining had M2 segment occlusions.

Patients with secondary MeVOs were younger (median age 75 versus 76 years; *p* = 0.031), had more severe stroke symptoms at baseline (median NIHSS 14 versus 10; *p* < 0.001), more often had received IVT (66% vs. 40%; *p* < 0.001), and were less often current smokers (7% versus 11%; *p* = 0.038), compared to patients with primary MeVO. There was a higher incidence of any postoperative intracranial hemorrhage in the secondary MeVO group (primary 23% vs. secondary 36%; *p* < 0.001) but no statistically significant differences in the incidence of sICH (3.3 and 4.3%; *p* = 0.219). Baseline data are shown in Table 1 and distribution of procedure related factors and postprocedural outcomes are shown in Table 2.

**Table 1:** Demographics, clinical presentation, and medical treatment for patients treated with endovascular thrombectomy for primary and secondary medium vessel occlusion (MeVO) in the middle cerebral artery (MCA)

	Primary MCA MeVO (*N* = 819)	Secondary MCA MeVO (*N* = 299)	Total + (*N* = 1118)	Missing
*Age, years (IQR)*	76 (68–82)	75 (66–81)	76 (68–82)	73 (7%)
*Female (%)*	354 (43%)	147 (49%)	501 (45%)	0 (0%)
Hypertension (%)	489 (60%)	182 (61%)	671 (60%)	77 (7%)
*Atrial fibrillation (%)*	377 (46%)	125 (42%)	502 (45%)	78 (7%)
*Diabetes Mellitus (%)*	169 (21%)	48 (16%)	217 (19%)	77 (7%)
*Current smoking (%)*	88 (11%)	20 (7%)	108 (10%)	270 (24%)
*Previous stroke (*%)	134 (16%)	34 (11%)	168 (15%)	76 (7%)
*Previous TIA (%)*	63 (8%)	20 (7%)	83 (7%)	78 (7%)
*NIHSS at baseline (IQR)*	10 (7–15)	14 (8.5–19)	11 (7–16)	19 (2%)
*Extracranial ICA occluded (%)*	25 (3%)	15 (5%)	40 (4%)	0 (0%)
*Intravenous thrombolysis (%)*	330 (40%)	198 (66%)	528 (47%)	8 (1%)
*Time of onset (%)*				1 (0%)
Known or estimated	750 (92%)	285 (95%)	1035 (93%)	
Unknown	68 (8%)	14 (5%)	82 (7%)	

Data presented as numbers (%) or medians with interquartile range (IQR)

*NIHSS* National Institute of Health Stroke Scale; *ICA* Internal Carotid Artery

**Table 2:** Distribution of procedure related factors and postprocedural outcomes after endovascular thrombectomy (EVT) in patients with primary and secondary medium vessel occlusion (MeVO) in the middle cerebral artery (MCA)

	Primary MCA MeVO (*N* = 819)	Secondary MCA MeVO (*N* = 299)	Total + (*N* = 1118)
*Process time (hh:mm)*			
Groin puncture <6 h	491 (60%)	215 (72%)	706 (63%)
Onset to groin puncture (IQR)	3:56 (2:37–7:12)	4:08 (2:42–5:31)	3:59 (2:38–6:28)
Onset to revascularization (IQR)	4:47 (3:23–8:09)	5:13 (3:32–6:47)	4:56 (3:27–7:31)
Groin to revascularization (IQR)	0:41 (0:27–1:05)	0:47 (0:30–1:15)	0:43 (0:28–1:07)
*Anesthesia (%)*			
General anesthesia (GA)	372 (45%)	94 (31%)	466 (42%)
Conscious sedation (CS)	428 (52%)	195 (65%)	623 (56%)
Converted from CS to GA	18 (2%)	10 (3%)	28 (3%)
*Treatment strategies (%)*			
Distal aspiration only	160 (19%)	36 (12%)	196 (17%)
Stent-retriever with/without direct aspiration	609 (74%)	237 (79%)	846 (76%)
Other	21 (3%)	13 (4%)	34 (3%)
*Revascularization (%)*			
mTICI 0–2a or EVT not possible	116 (14%)	52 (17%)	168 (15%)
mTICI 2b	207 (25%)	106 (35%)	313 (28%)
mTICI 2c–3	496 (61%)	140 (47%)	636 (57%)
*Hemorrhage 24* *h post-EVT*			
Any hemorrhage	188 (23%)	107 (36%)	295 (26%)
sICH (ECASS III criteria)	27 (3%)	13 (4%)	40 (4%)
*△NIHSS at 24* *h (IQR)*	−4 (−8 to −1)	−5 (−9 to 0)	−5 (−8 to 0)
mRS 90 days after EVT*			
0–1	179 (22%)	56 (19%)	235 (21%)
2	245 (30%)	88 (29%)	333 (30%)
3	136 (17%)	56 (19%)	192 (17%)
4	88 (11%)	35 (12%)	123 (11%)
5	62 (8%)	25 (8%)	87 (8%)
Dead	109 (13%)	39 (13%)	148 (13%)

Data presented numbers (%) or medians with interquartile range (IQR). Process times are presented with the format hours and minutes (hh:mm)

*sICH* symptomatic Intracranial Hemorrhage; *mTICI* modified Thrombolysis in Cerebral Infarction; *△NIHSS* Difference in National Institutes of Health Stroke Scale score before and 24 h after EVT; *mRS* modified Rankin Scale

*Imputed data. Missing data was below 4% except for: onset to groin puncture (12%), onset/groin to revascularization (13%), △NIHSS (13%), and sICH (13%)

Patients that were not successfully revascularized had higher rates of any postoperative intracranial hemorrhage (*N* = 57/168, 34%) compared to successfully revascularized patients (*N* = 237/949, 25%; *p* = 0.015). The rates of sICH according to the ECASS III criteria were also higher among non-revascularized patients (*N* = 17/168, 10%) than in all revascularized patients (*N* = 23/949, 2%; *p* < 0.001). Similarly, postoperative hemorrhages were more common among non-revascularized patients with primary MeVOs whereas no statistically significant differences in postoperative hemorrhages were observed among patients with secondary MeVOs (Supplement table 1). No statistically significant differences in age, sex, NIHSS at admission, or IVT-treatment were seen between groups (Supplement table 1).

### Primary and Secondary Outcomes

There was a small difference in the observed proportion of good functional outcome (mRS 0–2) after 90 days between primary (*N* = 424/819, 52%) and secondary MeVOs (*N* = 144/299, 48%), but there was no significant difference in the odds of good functional outcome for secondary MeVOs compared to primary MeVOs in the univariate analysis (OR 0.86, CI 95% 0.65–1.14) or in the multivariate analyses (OR 0.93, CI 95% 0.67–1.30) adjusted for age, sex, M3 segment occlusion location, pre-stroke mRS, and baseline NIHSS, using multiple imputations. The corresponding analyses using the original dataset, without multiple imputations, showed similar results in univariate (OR 0.93, CI 95% 0.69–1.27) and multivariate analyses (OR 1.16, CI 95% 0.82–1.64). Furthermore, there was no significant mean difference in early neurological improvement (∆NIHSS 0.26, CI 95% −0.71 to 1.24), between primary and secondary MeVO groups, *t* (971) = 0.529, *p* = 0.597.

### Exploratory Analyses

For patients with *primary MeVOs,* successful revascularization (*N* = 703) was associated with higher odds for good 90-day functional outcome when compared to non-revascularized patients (*N* = 116), in univariate as well as in multivariate analyses (OR 3.77, CI 95% 2.28–6.24, *p* < 0.001; and OR 4.46, CI 95% 2.40–8.29, *p* < 0.001, respectively). Patients successfully revascularized for *secondary MeVOs* (*N* = 246) also had higher odds for a good functional outcome at 90-days compared to non-revascularized patients (*N* = 52), in the univariate (OR 2.49, CI 95% 1.21–5.14; *p* = 0.013) as well as in the multivariate analyses (OR 2.76, CI 95% 1.16–6.56, *p* = 0.021), as illustrated in Fig. 2c.

In the sensitivity analyses, where patients with sICH (*N* = 40) were excluded, successful recanalization remained associated with good functional outcomes (mRS 0–2) for primary (OR 4.05, CI 95% 2.13–7.71, *p* < 0.001) and secondary MeVO patients (OR 2.49, CI 95% 1.04–5.98, *p* = 0.040), adjusting for age, sex, M3 segment occlusion, pre-stroke mRS, and baseline NIHSS. In the analyses of patients with less severe stroke symptoms (NIHSS ≤11; 63% mRS 0–2), the positive association between recanalization and good functional outcome remained for patients with primary MeVOs (OR 3.54, CI 95% 1.88–6.66, *p* < 0.001), whereas the results for the secondary MeVOs were non-significant, although showing a similar trend (OR 2.35, CI 95% 0.85–6.45, *p* = 0.098). However, the number of non-revascularized secondary MeVO patients was small (*n* = 22). Among patients with more severe baseline stroke symptoms (NIHSS >12; 37% mRS 0–2) revascularization increased the odds for good outcome for patients with primary MeVO (OR 4.88, CI 95% 1.85–12.8, *p* = 0.001) as well as secondary MeVO (OR 3.33, CI 95% 1.07–10.4, *p* = 0.038).

For both primary and secondary MeVOs successful revascularization was significantly associated with better ∆NIHSS, *primary*: mean difference 6.44 NIHSS points (CI 95% 5.03–7.84), *t* (709) = 8.962, *p* < 0.001, Cohen’s d = 1.001; *secondary*: mean difference 4.32 NIHSS points (CI 95% 2.09–6.56), *t* (260) = 3.795, *p* < 0.001, Cohen’s d = 0.616. The distribution in ∆NIHSS between the groups is illustrated in Fig. 2a. The results remained significant in the ANOVA with *p*-values <0.001 for both groups when including age and sex as covariates.

## Discussion

In this nationwide registry study of 5662 patients treated with EVT, 20% were treated for MCA MeVOs, of which 27% were secondary MeVOs. EVT for primary as well as secondary MCA MeVOs is associated with a good functional outcome at 90-days. Our study does not support the notion that the injuries from the initial large vessel occlusion offsets the benefits of revascularization of the MeVO [6, 7], which is in line with a previous meta-analysis of 14 smaller studies (with a total of 971 patients with primary distal MeVOs and 291 patients with secondary MeVOs) [8]. These results highlight the importance of revascularization for improving stroke outcomes for patients with primary as well as secondary MeVOs.

The rates of good functional outcomes (mRS 0–2) in this study (primary MeVO 52% and secondary MeVO 46%) are in line with a previous study [8]. Additionally, our exploratory analysis indicate that revascularization benefits patients with primary occlusions and less severe stroke symptoms (NIHSS ≤11), as well as patients with more severe (NIHSS >12) irrespective of primary or secondary status. The secondary MeVO group with less severe baseline stroke symptoms was small but showed a similar trend.

Our study suggests that patients with secondary MeVO have more severe neurological deficits at presentation than patients with primary MeVOs. However, early improvement in symptom severity after EVT was similar between primary and secondary MeVO groups (median NIHSS improvement of −4 versus −5) suggesting that the treatment effect is of the same magnitude.

Patients with secondary MeVOs were more likely to receive IVT prior to EVT compared to patients with primary MeVOs (66% versus 40%) but we cannot assess if the IVT therapy was completed, halted, or continued during the EVT procedure. The higher IVT rates may have contributed to the numerically higher incidence of any postoperative intracranial hemorrhage in patients with secondary occlusions (35.8% versus 22.9%) which however did not translate into higher rates of sICH. The preselection for IVT may have favorably impacted outcomes in the secondary MeVO group, as healthier patients are more likely to receive IVT and thereby improving the odds for a good functional outcome. There were no differences in IVT rates between patients that were successfully revascularized and non-revascularized patients. Despite this, higher rates of any postoperative hemorrhage were observed among non-revascularized patients. In our sensitivity analyses where we excluded patients with symptomatic ICH, successful recanalization remained associated with good functional outcomes for primary and secondary MeVO patients, therefore the observed effect is not likely caused by perioperative complications such as vessel perforations. A more likely cause is that non-revascularized patients suffered larger infarcts and therefore had an increased risk of postoperative symptomatic hemorrhage [16, 17].

Secondary MeVOs are inherently difficult to classify, and we must acknowledge that whatever categories we use, we will risk misinterpreting a substantial number of patients [4]. For example, our categorization did not allow us to capture infarction outside the parenchyma supplied by the occluded medium MCA-segment, such as frontal MCA territory infarction and occlusion of the inferior MCA trunc, or ACA territory infarct and M2-occlusion. Additionally, we excluded patients where the original clot had scattered causing multiple distal occlusions since these are even more difficult to categorize due to the large number of potential variants. However, we need to acknowledge that this limits the generalizability of our findings to patients where a single clot has moved distally.

### Limitations

In our study, we recognize several limitations. First, our data originates from nationwide quality registers where parameters like mTICI scores and postoperative complications are reported by the performing center without external validation which could introduce bias and potentially overestimate successful recanalization rates [18]. Nevertheless, the 84.9% successful recanalization rate in our study (mTICI 2b–3) is similar to what has been previously reported in other MeVO studies [19]. Secondly, 25% of the included patients that were known to be alive at 90 days, lacked follow-up mRS data. This may be attributable to patients with higher dependency being less likely to complete the follow-up questionnaire [20]. To address the missing outcome data, multiple imputations with predictive values were applied. However, the potential for attrition bias remains, which may have led to an overestimation of favorable outcomes. Thirdly, we compare patients with successful and unsuccessful revascularization in the exploratory analyses as an assessment of the treatment effect. While non-revascularized patients with large vessel occlusions have outcomes comparable to those receiving the best medical treatment in previous studies [21], we cannot exclude that our non-revascularized MeVO patients may have been negatively affected by the EVT procedure. However, our analyses do not support this, as non-revascularized patients showed unchanged postoperative symptom severity on a group level (median ∆NIHSS 0, see Supplement Table 1). It is also important to acknowledge that unsuccessfully revascularized patients may differ from the broader MeVO population, as they were selected by the treating physician for EVT based on the potential benefit of treatment. Even though our study represents real-world practice, the preselection for EVT treatment might have introduced a selection bias likely favoring patients with more severe neurological deficits and less severe comorbidities. Fourthly, our material did not allow us to separate proximal occlusions in dominant M2-branches from occlusions in more distal or non-dominant M2-branches. The efficacy of EVT may differ between these types of occlusions. We addressed this by performing exploratory analyses using baseline NIHSS as a surrogate for occlusion location which were in line with the main results. This has its inherent limitations as NIHSS may depend on the occluded territory or collateralization status, not solely on the proximal or distal clot location, and while higher NIHSS might reflect a more proximal occlusion on a group level this might not necessarily be true for an individual patient. However, our NIHSS cut-off of 11 corresponds well with the pooled data from 2 multicenter prospective cohort studies where the upper interquartile range cut-off for baseline NIHSS was 11.5 among patients with MeVOs, after excluding proximal M2 occlusions [22]. Future post-hoc analyses of the ongoing randomized controlled trials could be considered to evaluate efficacy of EVT for secondary distal MeVO.

## Conclusions

In this nation-wide high coverage registry study, patients with primary as well as patients with a single secondary MeVOs in the MCA territory have similar outcomes after EVT, and both groups seems to benefit from recanalization. These results indicates that EVT should not be withheld from MeVO patients due to primary or secondary MeVO type.

We would like to thank Åke Holmberg at EVAS and Fredrik Jonsson at Riksstroke for providing of data.

## Supplementary Information

Supplementary table 1. Distribution of baseline characteristics and treatment related factors between successfully revascularized patients (mTICI 2b-3) and non-revascularized patients
