# Supplementary material for: Outcomes After Thrombectomy for Primary and Secondary Medium Vessel MCA Occlusions: a Nationwide Registry Study
Source: Clin Neuroradiol. 2025 Mar 31;35(3):541–9. doi: 10.1007/s00062-025-01511-w (PMC12454531; doi:10.1007/s00062-025-01511-w)
Supplement: Supplementary file 1 — Supplementary table 1. Distribution of baseline characteristics and treatment related factors between successfully revascularized patients (mTICI 2b-3) and non-revascularized patients [file 62_2025_1511_MOESM1_ESM.docx]

**Supplementary Information**

**Title:** Outcomes after thrombectomy for primary and secondary medium vessel MCA occlusions: a nationwide, prospective registry study

**Journal:** *Clinical Neuroradiology*

**Authors:** Björn M. Hansen M.D., PhD.^1,2^, Emma Hall M.D.^1,2^, Birgitta Ramgren M.D., PhD.^1,2^, Teresa Ullberg M.D., PhD.^3,4^, Johan Wassélius M.D., PhD.^1,2^

**Affiliations:**

1. Department of Medical Imaging and Physiology, Skåne University Hospital, Lund, Sweden

2. Department of Clinical Sciences Lund, Radiology, Lund University

3. Department of Neurology, Skåne University Hospital, Malmö, Sweden

4. Department of Clinical Sciences Lund, Neurology, Lund University

**Corresponding author:**

Björn M Hansen M.D. PhD.
Email: [bjorn.hansen@med.lu.se](mailto:bjorn.hansen@med.lu.se)

| **Supplementary table 1.** Distribution of baseline characteristics and treatment related factors between successfully revascularized patients (mTICI 2b-3) and non-revascularized patients. | | | |
| --- | --- | --- | --- |
|  | **Revascularized** | **Non-revascularized** | **p-value** |
| **All patients, N= 1118** | **N= 949 (85%)** | **N= 168 (15%)** |  |
| Age (IQR) | 76 (68-82) | 76 (67-83) |  |
| Female (%) | 421 (44%) | 79 (47%) |  |
| NIHSS before EVT (IQR) | 11 (7-16) | 12 (7-17) |  |
| △NIHSS at 24 h (IQR) | -5 (-9 to -2) | 0 (-3.25 to 4.25) | <0.001 |
| Stroke onset to groin puncture, hh:mm (IQR) | 3:56 (2:39-6:19) | 4:20 (2:35-7:05) |  |
| Intravenous thrombolysis (%) | 447 (47%) | 80 (48%) |  |
| Any post-operative ICH (%) | 237 (25%) | 57 (34%) | 0.015 |
| sICH (ECASS III criteria) | 23 (2%) | 17 (10%) | <0.001 |
| **Primary MeVO, N = 819 (73%)** | **N = 703 (86%)** | **N = 116 (14%)** |  |
| Age (IQR) | 76 (68-82) | 77 (67.5-83) |  |
| Female (%) | 300 (43%) | 54 (47%) |  |
| NIHSS before EVT (IQR) | 10 (6-15) | 11 (7-15) |  |
| △NIHSS at 24 h (IQR) | -5 (-8 to -2) | 1 (-2 to 5) | <0.001 |
| Stroke onset to groin puncture, hh:mm (IQR) | 3:51 (2:37-6:48) | 4:25 (2:31-7:49) |  |
| Intravenous thrombolysis (%) | 281 (40%) | 49 (42%) |  |
| Any post-operative ICH (%) | 152 (22%) | 36 (31%) | 0.026 |
| sICH (ECASS III criteria) | 14 (2%) | 13 (11%) | <0.001 |
| **Secondary MeVO, N = 299 (27%)** | **N = 246 (83%)** | **N = 52 (17%)** |  |
| Age (IQR) | 75 (66-81) | 75.5 (64.75-81.25) |  |
| Female (%) | 121 (49%) | 25 (48%) |  |
| NIHSS before EVT (IQR) | 14 (9-18.75) | 15 (8-21) |  |
| △NIHSS at 24 h (IQR) | -6 (-10 to -1) | -0.5 (-5.25 to 4) | <0.001 |
| Stroke onset to groin puncture, hh:mm (IQR) | 4:09 (2:44-5:29) | 4:00 (2:35-5:35) |  |
| Intravenous thrombolysis (%) | 166 (67%) | 31 (60%) |  |
| Any post-operative ICH (%) | 85 (35%) | 21 (40%) | 0.425 |
| sICH, ECASS III criteria (%) | 9 (4%) | 4 (8%) | 0.247^a^ |
| Data presented numbers (%) or medians with interquartile range (IQR). Statistical analyses were performed with Mann-Whitney U-test, Pearson’s χ2 test, or ^a^Fisher’s exact test when rates or medians indicated possible relevant group differences. EVT, Endovascular Thrombectomy; MeVO, Medium Vessel Occlusion; sICH, symptomatic Intracranial Hemorrhage; △NIHSS, Difference in National Institutes of Health Stroke Scale score before and 24 hours after EVT. | | | |
